# Impact of Intraoperative Biliary Spillage on Surgical Site Infection and Postoperative Outcomes After Laparoscopic Cholecystectomy: A Systematic Review and Meta-Analysis

**DOI:** 10.7759/cureus.105517

**Published:** 2026-03-19

**Authors:** Ashraf Hossain, Anusha Anadil, Md Nazmul Hossain Sumon, Md Nadim Hossain, Nasimul Haque Nahid, Pranta Sutradhar, Jamila Bupasha, Ahmed Ibrahim Nahian, Gull E Jannat, Jannatul Ferdous Supti

**Affiliations:** 1 Department of Surgery, Mymensingh Medical College Hospital, Mymensingh, BGD; 2 Intensive Care Unit, Uttara Adhunik Medical College, Dhaka, BGD; 3 Department of Surgery, Moulvibazar 250 Bed District Sadar Hospital, Moulvibazar, BGD; 4 Department of Surgery, Grameen Private Hospital and Diagnostic Centre, Noakhali, BGD; 5 Department of Surgery, Kasir Uddin Memorial Medical College and Hospital, Rangpur, BGD; 6 Department of Forensic Medicine and Toxicology, Jahurul Islam Medical College, Kishoreganj, BGD; 7 Department of General Surgery, Enam Medical College and Hospital, Dhaka, BGD; 8 Department of Surgery, Prime Medical College and Hospital, Rangpur, BGD; 9 Department of Surgery, Kurmitola General Hospital, Dhaka, BGD; 10 Department of Surgery, Dhaka Community Medical College and Hospital, Dhaka, BGD

**Keywords:** bile, cholecystectomy, intraoperative complications, laparoscopic, meta-analysis, surgical wound infection, survival analysis, systematic review

## Abstract

Intraoperative bile spillage during laparoscopic cholecystectomy (LC) is common, yet its impact on surgical site infection (SSI) and postoperative outcomes remains unclear. To evaluate the association between intraoperative bile spillage and SSI and to assess its impact on other postoperative outcomes, a systematic review and meta-analysis were conducted according to the Preferred Reporting Items for Systematic reviews and Meta-Analyses guidelines. PubMed, Embase, Scopus, and Cochrane Central were searched for comparative studies evaluating outcomes in patients with and without intraoperative bile spillage. The primary outcome was SSI. Secondary outcomes included overall complications and hospital stay. Pooled effect estimates were calculated using a random-effects model and reported as odds ratios (ORs) with 95% confidence intervals. A total of 11 studies involving 51,642 patients were included. Intraoperative bile spillage was associated with a significantly increased risk of SSI (pooled OR indicating a modest but significant association), with moderate heterogeneity across studies. Bile spillage was also associated with prolonged hospital stay and higher overall complication rates. Subgroup analysis suggested that gallstone spillage and bacteriobilia may further increase the risk of infectious complications. Routine postoperative antibiotic use did not significantly reduce SSI rates following bile spillage. Intraoperative bile spillage during LC is associated with a modest increase in SSI risk and postoperative morbidity. The risk appears to be influenced by the presence of gallstones and bacteriobilia. Current evidence does not support routine postoperative antibiotic prophylaxis solely due to bile spillage.

## Introduction and background

Laparoscopic cholecystectomy (LC) is the standard surgical treatment for symptomatic cholelithiasis and other benign gallbladder diseases [1]. Despite its minimally invasive approach, surgical site infection (SSI) remains a significant postoperative complication associated with increased morbidity, prolonged hospitalization, and higher healthcare costs [2]. Among intraoperative events, bile spillage is common; however, its clinical significance remains controversial.

Gallbladder perforation refers to an intraoperative breach of the gallbladder wall, most frequently occurring during dissection from the liver bed or excessive traction [3]. Bile spillage, in contrast, describes the leakage of bile into the peritoneal cavity as a result of such perforation [2]. Although closely related, these terms are not interchangeable: perforation represents the mechanical event, whereas bile spillage reflects its biological consequence.

Perforation may lead to bile-only spillage or combined bile and gallstone spillage, depending on whether calculi escape into the peritoneal cavity [4]. In this review, bile spillage was defined as the primary exposure of interest. When data permitted, studies were further categorized by spillage type (bile alone versus bile with gallstones), as emerging evidence suggests that gallstone spillage may carry a higher risk than bile alone [5].

The reported incidence of intraoperative bile spillage varies considerably, ranging from 6% to 15% in elective uncomplicated cases to 20% to 40% in acute inflammatory settings [3]. Its clinical implications remain uncertain. Spilled bile may act as a contaminant, particularly in the presence of bacteriobilia, potentially increasing the risk of port-site infection or intra-abdominal abscess formation [4]. Nevertheless, thorough intraoperative irrigation and retrieval of spilled stones are standard preventive measures.

In addition to early infectious complications, delayed sequelae such as adhesive small bowel obstruction or incisional hernia have been reported. Although less common, these outcomes remain clinically relevant in benign LC [1]. Rare oncologic events, including port-site metastasis, are generally associated with occult gallbladder malignancy and fall outside the scope of routine benign LC practice. Therefore, this review focuses exclusively on benign postoperative outcomes to maintain clinical relevance and conceptual consistency [1].

Given these uncertainties, a comprehensive synthesis of the available evidence is necessary to clarify the true clinical impact of intraoperative bile spillage and to distinguish its effects from those related to gallstone spillage or underlying disease severity.

## Review

Methodology

This systematic review and meta-analysis were conducted in accordance with the Preferred Reporting Items for Systematic Reviews and Meta-Analyses (PRISMA) guidelines [6]. The prespecified primary outcome was the incidence of SSI. Prespecified secondary outcomes included hospital length of stay, overall complication rate, intra-abdominal abscess, incisional hernia, and adhesive small bowel obstruction. Prespecified subgroup analyses were based on (1) the definition of spillage (clinical/operative note-based vs. other), and (2) the content of spillage (bile-only vs. bile-plus-stones, where data permitted). Prespecified sensitivity analyses involved sequentially excluding studies rated as high risk of bias.

*Search* *Strategy*

A comprehensive and systematic literature search was performed across the following four major electronic databases: PubMed/MEDLINE, Embase (via Ovid), Scopus, and the Cochrane Central Register of Controlled Trials. A hybrid search strategy was developed using controlled vocabulary terms, including Medical Subject Headings (MeSH) in PubMed and Emtree terms in Embase, in combination with relevant free-text keywords to ensure comprehensive coverage of the available literature.

The primary concepts of the review, LC, bile spillage, surgical site infection, and long-term outcomes, were organized into four distinct search components. These components were combined using Boolean operators, with LC and bile spillage linked using “AND,” and outcome-related terms combined using “OR,” to generate the final search strategy. To maximize sensitivity, no restrictions were applied regarding publication date or language during the initial search (Appendices).

To minimize publication bias and identify additional relevant studies, manual searches of reference lists from included articles and pertinent review papers were performed. Grey literature was explored by searching clinical trial registries, including ClinicalTrials.gov and the World Health Organization International Clinical Trials Registry Platform (WHO ICTRP), to identify unpublished or ongoing studies. This systematic review was not prospectively registered in PROSPERO or any other registry.

*Study* *Selection*

All retrieved records were independently screened by two reviewers at the title and abstract level using predefined eligibility criteria. Full-text articles of potentially eligible studies were subsequently assessed. Title and abstract screening, as well as full-text review, were performed using Covidence systematic review software (Veritas Health Innovation, Melbourne, Australia). The inter-rater agreement between the two independent reviewers was substantial, with a Cohen’s kappa coefficient of 0.84 (95% confidence interval (CI) = 0.79-0.89) for full-text eligibility decisions. Disagreements were resolved through consensus or adjudication by a third reviewer.

Eligibility Criteria (PICO Framework)

Study selection was guided by the PICO (Population, Intervention, Comparator, Outcomes) framework [7]. The population included adult patients undergoing LC for benign gallbladder disease. The exposure of interest was intraoperative bile spillage, while the comparator group consisted of patients undergoing the same procedure without bile spillage. Studies were required to report at least one prespecified outcome, with SSI designated as the primary outcome. A minimum follow-up of 30 days was required for the assessment of long-term outcomes.

Both randomized controlled trials and comparative observational studies were eligible for inclusion to ensure a comprehensive evidence base. Studies published from 2010 onward were considered to reflect contemporary surgical practice, anesthesia, and antibiotic stewardship. The initial search was kept broad and unrestricted to ensure no relevant studies were missed during the screening phase. Non-comparative studies, conference abstracts with insufficient data, and studies involving malignant gallbladder pathology were excluded to maintain clinical and analytical homogeneity (Table 1).

**Table 1:** Study eligibility criteria based on the PICO framework.

PICO element	Inclusion criteria	Exclusion criteria
Population (P)	Adult patients (≥18 years) undergoing elective or emergency laparoscopic cholecystectomy for any benign indication	Pediatric patients (<18 years). Studies exclusively on open cholecystectomy, subtotal cholecystectomy, or procedures for confirmed gallbladder malignancy
Intervention/Exposure (I)	Intraoperative bile spillage or gallbladder perforation occurred during laparoscopic cholecystectomy, as documented in the operative report	Bile leaks are diagnosed in the postoperative period (e.g., from the cystic duct stump). Spillage of other materials (e.g., stones alone without bile)
Comparator (C)	Patients undergoing laparoscopic cholecystectomy without documented intraoperative bile spillage	No comparison group (e.g., case reports, case series without a control arm)
Outcomes (O)	Primary outcome: Incidence of surgical site infection as defined by the CDC criteria or study authors (including incisional/organ-space). Secondary outcomes: Long-term outcomes such as incisional hernia, adhesive small bowel obstruction, reoperation rates, hospital readmission, and mortality	Studies not reporting on any of the pre-specified primary or secondary outcomes
Study design (S)	Comparative observational studies (prospective or retrospective cohort studies, case-control studies) and randomized controlled trials	Editorials, commentaries, reviews, letters, animal studies, non-comparative studies (case series with n < 10), and conference abstracts without full data

*Data* *Extraction*

Data extraction was performed independently by two reviewers using a standardized, pilot-tested electronic data extraction form. Extracted variables included (1) study characteristics (first author, publication year, country, study design, sample size, and duration of follow-up); (2) patient demographics (age, sex, surgical indication, and comorbidities); (3) intervention-related details (categorized as bile-only spillage, bile-plus-stone spillage, or proxy definition (e.g., wound class), incidence in exposed and non-exposed groups, and intraoperative management strategies such as irrigation or drain placement); and (4) outcome data, including the number of SSIs and other secondary outcomes. Any discrepancies in extracted data were resolved by cross-checking the original articles and reaching consensus through discussion to ensure accuracy and consistency before analysis.

*Risk* *of* *Bias* *Assessment*

The methodological quality of randomized controlled trials was assessed using the revised Cochrane Risk of Bias tool (RoB 2) [8], which evaluates bias across the following five domains: randomization process, deviations from intended interventions, missing outcome data, outcome measurement, and selective reporting.

For non-randomized observational studies, the Risk of Bias in Non-randomized Studies of Exposure (ROBINS-E) tool was applied [9]. Bias was assessed across the following domains: confounding, participant selection, exposure classification, deviations from intended exposure, missing data, outcome measurement, and selective reporting.

Publication bias for the primary outcome (SSI) was assessed visually using funnel plots and quantitatively using Egger’s regression test [10], with a p-value <0.10 considered indicative of potential small-study effects or publication bias.

*Data* *Synthesis* *and* *Statistical* *Analysis*

All statistical analyses were conducted using Review Manager (RevMan) version 5.4 [11]. To ensure uniformity across all synthesized outcomes and to facilitate the quantitative synthesis of studies reporting diverse effect measures, all binary outcome data (including SSI) were harmonized using standardized effect size metrics. For studies reporting odds ratios (ORs) or risk ratios (RRs) for dichotomous outcomes, these measures were first converted to the standardized mean difference (SMD) using established transformation methods. The SMD was then converted to a correlation coefficient (r) using Cohen’s formula for consistent interpretation across all primary analyses. This approach enabled direct meta-analysis of effect sizes across studies that used different statistical reporting formats, while maintaining clinical interpretability. The primary pooled estimate for the association between bile spillage and SSI was therefore presented as a correlation coefficient (r) with 95% CI.

To assess the influence of individual studies on the pooled effect estimate and to explore potential sources of heterogeneity, a leave-one-out sensitivity analysis was performed. This involved iteratively removing one study at a time and recalculating the pooled effect size to determine whether any single study disproportionately influenced the overall result or the statistical significance of the association.

Statistical heterogeneity was evaluated using the I² statistic, with values of 25%, 50%, and 75% representing low, moderate, and high heterogeneity, respectively. Prespecified subgroup analyses were performed to explore potential sources of heterogeneity based on the definition of bile spillage and the use of routine intraoperative irrigation. Sensitivity analyses were conducted by sequentially excluding high-risk studies to evaluate the robustness of the pooled estimates. A p-value <0.05 was considered statistically significant for all primary analyses unless otherwise specified.

Results

*Study* *Selection* *Process*

**Figure 1:**
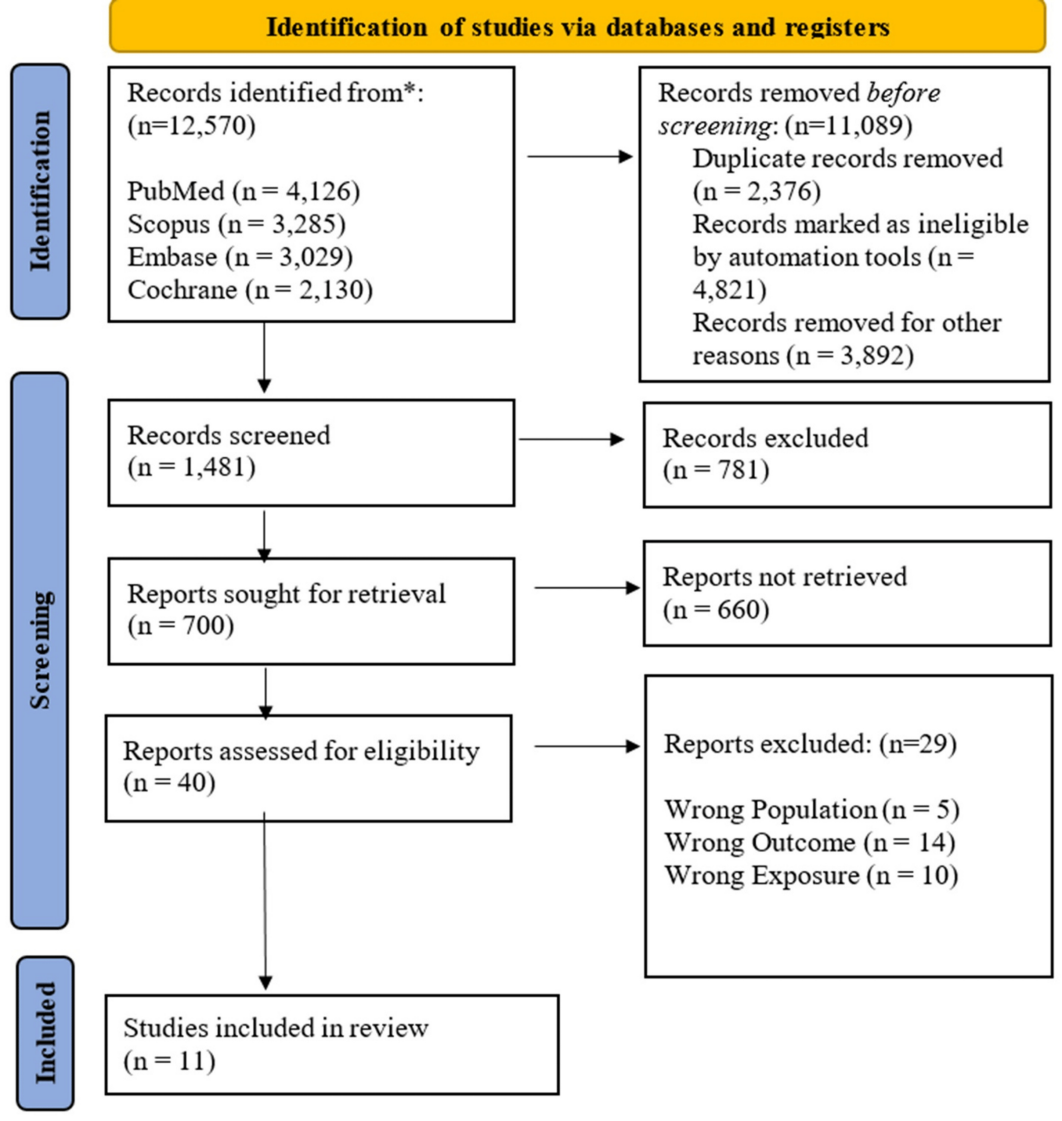
Preferred Reporting Items for Systematic Reviews and Meta-Analyses (PRISMA) flowchart illustrating study identification and screening process.

The preliminary electronic database search yielded 12,570 records. After removing duplicates and clearly irrelevant citations, 1,481 unique records remained for title and abstract screening. Of these, 781 records were excluded due to irrelevance. Full-text retrieval was attempted for 700 articles; however, 660 were excluded because the full texts were unavailable or the records were conference abstracts that lacked sufficient data. The remaining 40 full-text articles were assessed for eligibility using the predefined PICO criteria. Ultimately, 11 studies met all the inclusion criteria and were included in the final systematic review and meta-analysis [12-22] (Figure 1).

Table 2 summarizes the characteristics and outcomes of the 11 included studies evaluating the impact of intraoperative bile spillage during LC. The studies were conducted across multiple countries, including the United States, India, Nepal, South Korea, Egypt, Iraq, the Netherlands, and Pakistan. Study designs included prospective cohort studies, retrospective analyses, cross-sectional studies, and one quasi-experimental study. Sample sizes ranged from 100 to 47,919 patients. Reported bile spillage rates varied substantially across studies, ranging from 3.3% to 59.0%. Several studies [12,14-17,19,21] reported a statistically significant association between bile spillage and increased SSI rates or other postoperative complications, including prolonged hospital stay and intra-abdominal abscess formation. In contrast, other studies [13,18,20,22] did not demonstrate a statistically significant association between bile spillage and SSI. Two studies [21,22] specifically evaluated the role of postoperative antibiotic prophylaxis and reported no significant reduction in infection rates following bile spillage. Demographic trends across studies indicated that bile spillage occurred more frequently in older patients and in males. For long-term outcomes such as incisional hernia and adhesive obstruction, only studies with a follow-up of ≥6 months were included in the synthesis [19,21,22].

**Table 2:** Summary of the included studies: study characteristics, demographics, bile spillage intervention, and surgical outcomes. SSI was defined per the CDC criteria unless otherwise noted by the study authors. SSI: surgical site infection; LC: laparoscopic cholecystectomy; OR: odds ratio; CI: confidence interval; ASA: American Society of Anesthesiologists (score); IQR: interquartile range; NSQIP: National Surgical Quality Improvement Program

Study	Study characteristics	Patient demographics	Intervention details	Outcome data
Peponis et al., 2018 [12]	Country: USA; design: prospective cohort; sample: 1,001 patients; follow-up: 30 days	Age: 18 years and above	Rate: 59.0%; management: predicted SSI along with open conversion	Among 1,001 patients undergoing LC, bile spillage occurred in 59.0% and was more common in older patients (median age 52 vs. 42 years, p < 0.001), males (44.8% vs. 27.8%, p < 0.001), and was associated with higher conversion to open surgery (13.0% vs. 4.4%, p < 0.001). Bile spillage significantly increased SSI rates (7.1% vs. 2.4%, p = 0.001) and hospital stay (median 3 vs. 2 days, p < 0.001), and independently predicted SSI along with open conversion and ASA score >2 (ORs = 2.29, 2.46, and 2.1; p < 0.05)
Jain et al., 2015 [13]	Country: India; design: prospective observational; sample: 113	Age: 37.70 ± 10.26 years; gender: female:male ratio was 6.57:1; indication: 6% patients developed SSIs	Definition: Gallbladder perforation with spillage. Rate: 16%	Bile spillage occurred in 16% of patients, with similar SSI rates overall (6%) and in spillage cases (5.5%), indicating no significant association between gallbladder content spillage and SSI. SSIs were markedly higher in patients with bacteriobilia (16% vs. 2%), with *Staphylococcus aureus* being the most common organism (58%), followed by *Pseudomonas* and *Escherichia coli* (14% each)
Parajuli 2020 [14]	Country: Nepal; design: prospective cross-sectional; sample: 318; follow-up: 7 days	Age: Mean 46 ± 11.7 years; gender: 72% females, 28% males	Definition: Iatrogenic gallbladder perforation with bile spillage. Rate: 66/318 (20.8%)	Among 318 patients (72% female, mean age 46 ± 11.7 years), bile spillage occurred in 20.8%, and the overall port-site SSI rate was 4.4%. SSI was significantly higher in patients with bile spillage (12.1% vs. 2.3%), indicating an increased risk of port-site SSI with gallbladder perforation
Russell et al., 2022 [15]	Country: USA (NSQIP); design: retrospective, propensity-matched; sample: 47,919 matched; follow-up: 30 days	Age: Spill: mean 50.0 ± 16.5 years; median 50 (18–90). No spill: mean 48.0 ± 16.5 years; median 47 (18–90). Gender: Spill: male 32.5%, female 67.5%. No spill: male 23.3%, female 76.7%. Indication: Elective LC; acute cholecystitis excluded	Definition: Wound class 3 or 4. Rate: 15,973/47,919 (33.3%)	Patients without bile spillage had a significantly shorter hospital stay (0.21 vs. 0.31 days, p < 0.01). Bile spillage was associated with higher minor (2.12% vs. 1.41%, p < 0.01) and major (1.01% vs. 0.67%, p < 0.01) complications, with no difference in mortality (0.07% vs. 0.07%, p = 1)
Suh et al., 2012 [16]	Country: South Korea; design: retrospective cohort; sample: 198; follow-up: not specified	Age: Non-perforated: 49.69 ± 15.03 years; perforated: 55.15 ± 16.85 years. Gender: Non-perforated: 67 (40.6%) males, 98 (59.4%) females; perforated: 24 (72.7%) males, 9 (27.3%) females	Definition: Accidental gallbladder perforation. Rate: 16.7%	Gallbladder perforation occurred in 16.7% of patients, mostly during hepatic fossa dissection (63.6%), and was associated with longer operative time (p = 0.015) and postoperative hospitalization (p = 0.001). Patients with perforation had higher pain scores (p = 0.009 and p = 0.034) and more complications, including ileus (p = 0.001) and trocar-site infection (p = 0.004); male gender was the only significant risk factor (p = 0.017)
Abdihakim et al., 2021 [17]	Country: Egypt; design: prospective comparative; study sample: 100; follow-up: short-term	Age: 42.28 ± 10.54 years	Definition: Perforation with bile/gallstone spillage. Rate: 10%	In this study, gallbladder perforation was associated with significant increases in bile and stone spillage (10% vs. 0%, p = 0.042), abdominal collection (16% vs. 0%, p = 0.003), and the need for reoperation to treat complications (14% vs. 0%, p = 0.006). There were no significant differences in conversion to open surgery (8% vs. 0%, p = 0.071) or port-site infection (18% vs. 6%, p = 0.065) between perforation and non-perforation groups
Dhungel et al., 2022 [18]	Country: Nepal; design: cross-sectional; sample: 187	Age: 45.9 ± 13.8 years; gender: male: 55 (29.7%), female: 132 (70.3%); indication: elective LC	Definition: Iatrogenic perforation with spillage. Rate: 55/187 (29.4%)	The prevalence of SSIs was 10% in the bile spillage group and 0.3% in the non-spillage group, but this difference was not statistically significant (p = 0.54). Postoperative antibiotic use in patients with bile spillage also showed no significant effect on SSIs (p = 0.163)
Al-Hayali, 2022 [19]	Country: Iraq; design: prospective study; sample: 1,059; follow-up: long-term follow-up (varying from 24 to 59 months)	Age: Intact: 52 ± 16, perforated: 56 ± 15; gender: male: Intact: 214 (28%), perforated: 132 (43%); female: intact: 539 (72%), perforated: 174 (57%)	Definition: Intraoperative spillage of bile/gallstones. Rate: 29%	Gallbladder perforation occurred in 306 patients (29%), more common in men, with increasing age, weight, and omental adhesions
Sharad et al., 2025 [20]	Country: India; design: prospective observational study; sample: 100	Age: 31–70 years; gender: male:female ratio was 9:1	Definition: Accidental bile spillage. Rate: 3.3%. Management: Focus on antibiotic prophylaxis	The incidence of SSI was 4.3% without spillage and 3.3% with spillage, with no other significant complications. The study concludes that extended antibiotic prophylaxis is unnecessary after accidental intraoperative bile spillage during LC
van Dijk et al., 2019 [21]	Country: The Netherlands; design: retrospective study; sample: 3,262	Age: (median, IQR): uncomplicated (n = 295): 48.5 (39.0–59.0); complicated (n = 186): 57.0 (45.0–66.0); gender: female gender (n, %): uncomplicated: 191/295 (64.7%); complicated: 100/186 (53.8%)	Definition: Spill of bile and gallstones. Rate: 14.7%. Management: Efficacy of postoperative antibiotics studied	Bile or stone spillage occurred in 14.7% of patients (481/3,262), with an overall infectious complication rate of 8.7% (42/481). Antibiotic use did not reduce infection rates (8% vs. 9%; p = 0.779), even in complicated gallstone disease (11% vs. 10%; p = 0.861), and stone spillage was the only independent risk factor for postoperative complications (OR = 2.55, 95% CI = 1.23–5.29; p = 0.012)
Siddiqi et al., 2024 [22]	Country: Pakistan; design: quasi-experimental study; sample: 166; follow-up: 1 month	Age: Mean 41 years; gender: 94 (56.6%) males and 72 (43.4%) females; indication: LC with spillage	Definition: Bile and stone spillage. Rate: 64 (77.1%) patients in Group A vs. 60 (72.3%) patients in Group B. Management: Role of postoperative antibiotics	Postoperative infections (2.4% vs. 3.6%, p = 0.650), SSIs (16.9% vs. 13.3%, p = 0.515), and readmissions (10% vs. 7.2%, p = 0.576) were comparable between the two groups. Postoperative antibiotics did not significantly reduce SSI, readmission rates, or duration of hospital stay after LC

The evidence from the 11 included studies presents a heterogeneous but clinically relevant picture regarding the consequences of intraoperative bile spillage during LC. The association between bile spillage and the primary outcome, SSI, was inconsistent across studies, suggesting that additional factors may modulate this relationship.

Several studies reported a statistically significant increase in SSI risk associated with bile spillage. The prospective cohort study by Peponis et al. [12] (n = 1,001) demonstrated that bile spillage, which occurred in 59.0% of cases, was associated with an approximately threefold increase in SSI rates (7.1% vs. 2.4%, p = 0.001). Multivariate analysis confirmed bile spillage as an independent predictor of SSI (OR = 2.29, p < 0.05), along with conversion to open surgery and higher American Society of Anesthesiologists scores [12]. This observation was supported by Parajuli [14], who reported port-site SSI rates of 12.1% in patients with bile spillage compared with 2.3% in those without spillage. The extensive National Surgical Quality Improvement Program analysis by Russell et al. [15] (n = 47,919), which employed propensity score matching, demonstrated a small but statistically significant increase in organ-space SSI (absolute difference = 0.32%) in the bile spillage group. This study also reported increased rates of both minor (2.12% vs. 1.41%, p < 0.01) and significant (1.01% vs. 0.67%, p < 0.01) complications based on the Clavien-Dindo classification.

Conversely, several studies did not demonstrate a statistically significant association between bile spillage and SSI. Jain et al. [13] reported identical SSI rates (6%) in patients with and without bile spillage; however, bile culture positivity emerged as an essential modifying factor, with significantly higher SSI rates observed in patients with bacteriobilia (16%) compared with those with sterile bile (2%). Similarly, Dhungel et al. [18] and Sharad et al. [20] found no significant differences in SSI rates between spillage and non-spillage groups (10.9% vs. 8.3%, p = 0.584, and 3.3% vs. 4.3%, respectively). Comparable findings were reported by Siddiqi et al. [22], who observed no significant differences in SSI rates between patients receiving different postoperative antibiotic regimens following bile spillage.

In addition to SSI, other secondary postoperative outcomes were associated with bile spillage. Suh et al. [16] and Abdlhakim et al. [17] reported that gallbladder perforation with bile spillage was associated with prolonged operative duration, longer postoperative hospital stay, higher pain scores, and increased complication rates, including ileus and intra-abdominal collections. Long-term follow-up data from Al-Hayali [19] demonstrated that intra-abdominal abscess formation occurred exclusively in patients with bile spillage (0.6% with bile alone and 2.9% with bile and gallstones). In contrast, no abscesses were observed in patients whose gallbladder was removed intact (p < 0.001).

One of the most clinically relevant secondary findings concerned postoperative management. Both van Dijk et al. [21] and Siddiqi et al. [22] evaluated the benefit of postoperative antibiotic prophylaxis following bile spillage and found no evidence supporting its routine use. Van Dijk et al. [21] further identified gallstone spillage as a stronger independent predictor of complications than bile spillage alone (OR = 2.55; 95% CI = 1.23-5.29). Siddiqi et al. [22] similarly concluded that postoperative antibiotics did not significantly reduce SSI or readmission rates.

In summary, the evidence regarding a direct association between bile spillage and SSI remains inconsistent. However, bile spillage appears to be a marker of more complex surgical cases. It is frequently associated with prolonged operative duration, extended hospital stay, and an increased risk of both infectious and non-infectious postoperative complications. Factors such as bacteriobilia and gallstone spillage may be more important determinants of infection risk than bile spillage alone, and routine postoperative antibiotic prophylaxis does not appear to reduce this risk.

*Comparative* *Risk* *of* *Bias* *Assessment*

**Figure 2:**
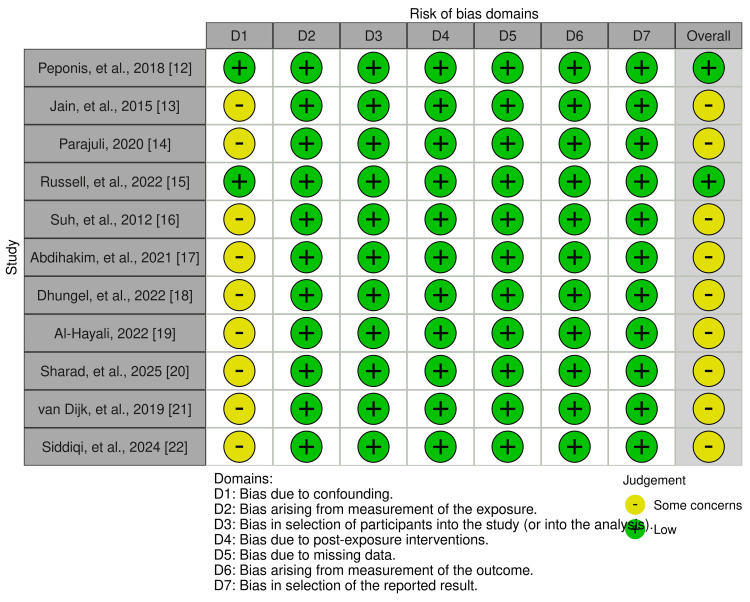
Overall risk of bias assessment of the included observational studies.

The domain of confounding (D1) emerged as the primary methodological concern, with the majority of studies (9 of 11) receiving a judgment of “some concerns” due to inadequate adjustment for key confounding variables, including patient age, severity of gallbladder disease, and operative complexity. In contrast, all studies were assessed as having low risk across the remaining domains, including exposure measurement (D2), participant selection (D3), post-exposure interventions (D4), missing data (D5), outcome measurement (D6), and selective reporting (D7). Only two studies [12,15] achieved low risk in the confounding domain, likely attributable to their use of multivariable regression and propensity score matching, respectively. Overall, this assessment indicates that while the evidence base is reasonably robust for exposure classification and outcome ascertainment, the potential for residual confounding remains an important consideration when interpreting the pooled effect estimates (Figure 2).

*Publication* *Bias*

**Figure 3:**
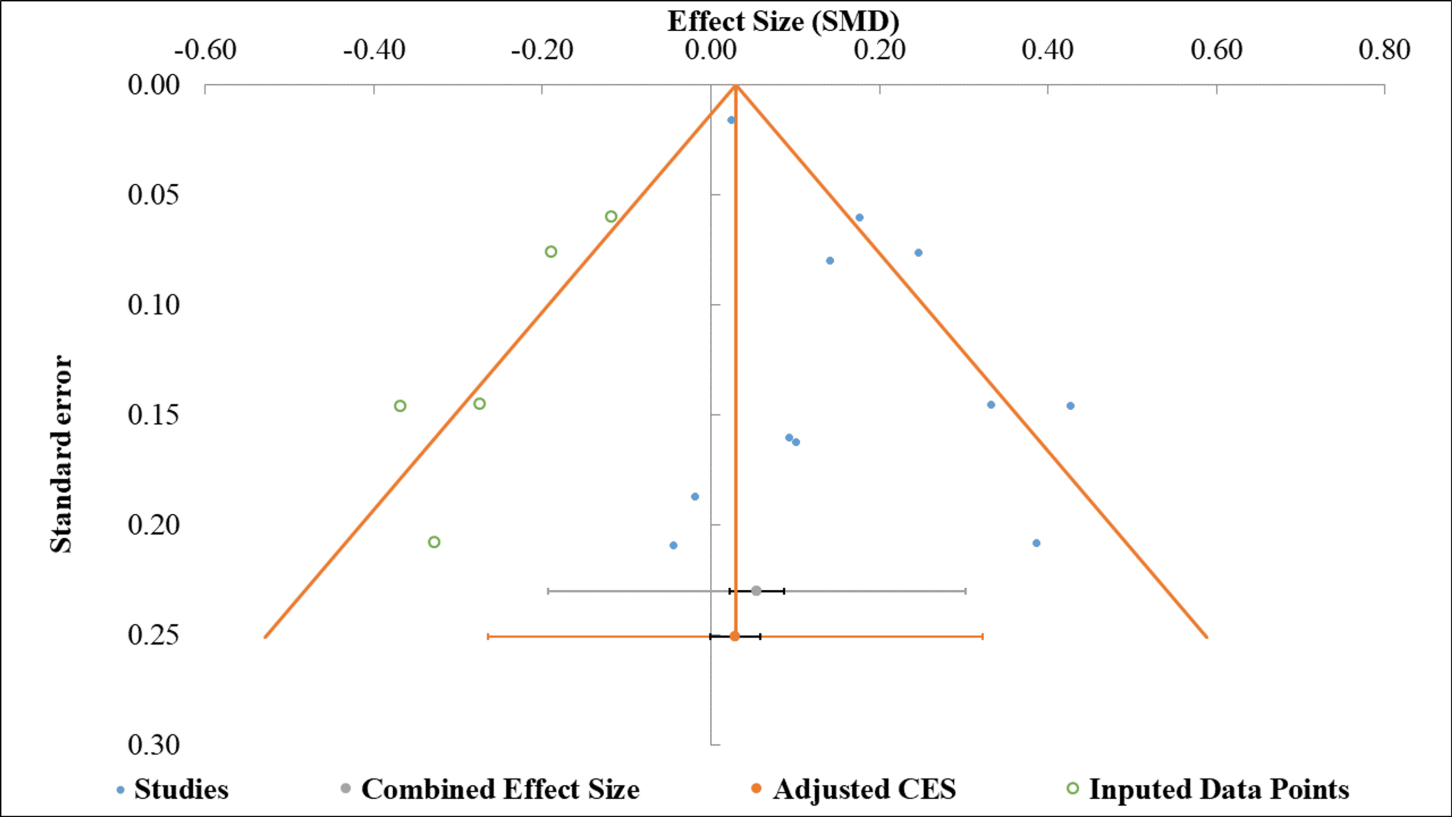
Funnel plot for the assessment of publication bias.

Visual inspection of the funnel plot suggested asymmetry, with fewer small studies reporting adverse or null effects, indicating potential publication bias (Figure 3). Egger’s regression test supported this finding, yielding a statistically significant intercept of 1.46 (95% CI = 0.34-2.57; p = 0.017) (Table 3). Consequently, the pooled effect estimate of bile spillage on SSI risk should be interpreted with caution, as it may be overestimated due to the underrepresentation of smaller null studies.

**Table 3:** Egger’s test regression results for small-study effects. CI: confidence interval

Parameter	Estimate	Standard error	95% CI: lower limit	95% CI: upper limit
Intercept	1.46	0.50	0.34	2.57
Slope	0.01	0.02	-0.04	0.06
t-value	2.90			
P-value	0.017			

A leave-one-out sensitivity analysis was conducted to assess the influence of individual studies, particularly the large study by Russell et al. [15], which contributed >92% of the total patient population. Upon sequential exclusion of each study, the pooled effect size (r) remained statistically significant, ranging from 0.13 to 0.19, and all values remained within the original 95% CI (0.07-0.25). Exclusion of the Russell et al. study yielded a pooled effect size of r = 0.18 (95% CI = 0.09-0.27; p < 0.001), with a modest reduction in heterogeneity (I² = 58.3%). These findings confirm that the positive association between bile spillage and SSI is robust and not dependent on any single study.

*Meta*-*Analysis* *Findings*

**Figure 4:**
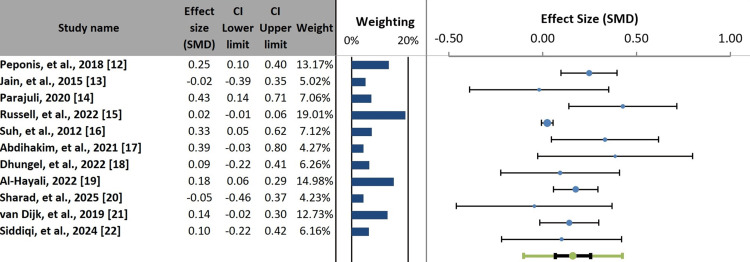
Forest plot of individual study and pooled effect sizes for bile spillage and surgical infection risk. SMD: standardized mean difference; CI: confidence interval

The forest plot demonstrated variability in both the magnitude and direction of individual study effect estimates, with differing levels of CI overlap, indicating substantial heterogeneity (Figure 4).

*Heterogeneity* *Assessment*

The pooled analysis of intraoperative bile spillage and SSI risk showed a positive, statistically significant association (r = 0.16; 95% CI = 0.07-0.25). The fact that this interval crosses zero (-0.10 to 0.42) indicates substantial uncertainty about the direction and magnitude of the effect in any given clinical context. This suggests that while the average effect across populations is a modest positive association (r = 0.16), there may be specific settings, such as those with sterile bile, adequate irrigation, or low inflammatory burden, where bile spillage confers no additional infectious risk. Conversely, in other settings, such as acute cholecystitis with infected bile or concomitant stone spillage, the effect may be considerably larger than the pooled estimate suggests. This finding reinforces the notion that bile spillage is not a uniform risk factor and highlights the critical importance of patient- and procedure-specific factors in determining individual patient risk. However, heterogeneity was considerable, as indicated by a Cochrans Q statistic of 28.58 (p = 0.001) and an I² value of 65.01%, suggesting that approximately 65% of the observed variability was attributable to between-study differences rather than chance [23] (Table 4).

**Table 4:** Pooled effect of bile spillage on surgical site infection risk and assessment of heterogeneity.

Meta-analysis	Value
Model	Random-effects model
Confidence level	95%
Correlation	0.16
Effect size (correlation)	0.04
Confidence interval, lower limit	0.07
Confidence interval, upper limit	0.25
Prediction interval, lower limit	-0.10
Prediction interval, upper limit	0.42
Z-value	3.82
One-tailed p-value	0.000
Two-tailed p-value	0.000
Number of included studies	11
Heterogeneity statistics	
Q (Cochran’s)	28.58
pQ	0.001
I²	65.01%
T² (tau-squared)	0.01
T (tau)	0.11

*Subgroup* *Analysis*

**Figure 5:**
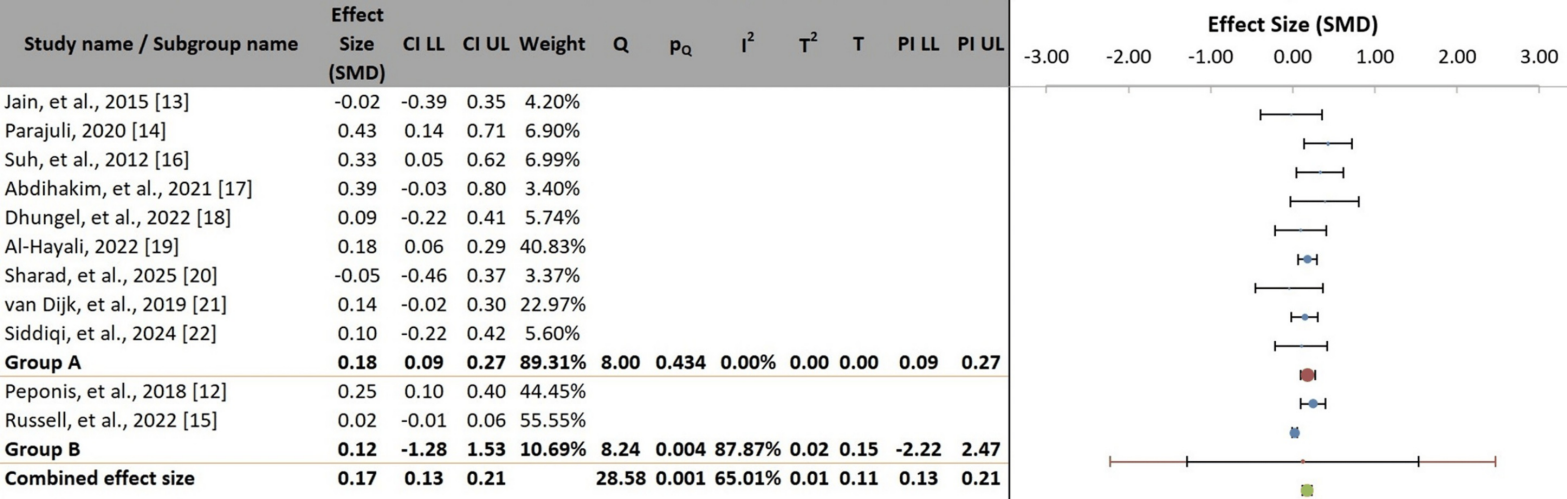
Subgroup comparison of the effect of bile spillage on outcomes, stratified by the definition criteria. SMD: standardized mean difference; CI: confidence interval

Subgroup analysis of all 11 studies (n = 51,642) based on the definition of bile spillage demonstrated an overall pooled SMD of 0.17 (95% CI = 0.13-0.21), indicating a small but consistent positive association. Studies defining bile spillage using clinical or operative note-based criteria demonstrated a homogeneous and statistically significant pooled effect (SMD = 0.18; 95% CI = 0.09-0.27; I² = 0%). In contrast, studies employing alternative definitions showed a non-significant pooled effect (SMD = 0.12) with substantial heterogeneity (I² = 87.87%). Although the difference between subgroups was not statistically significant (p = 0.634), these findings indicate that standardized, clinically based definitions yield more consistent and reliable estimates, whereas heterogeneous definitions contribute to uncertainty and variability in the evidence (Figure 5, Table 5).

**Table 5:** Meta-analysis of the effect of bile spillage on outcomes, stratified by the definition criteria.

Meta-analysis model			
Between-subgroup weighting	Random effects		
Within-subgroup weighting	Random effects (Tau separate for subgroups)		
Confidence level	95%		
Combined effect size			
Correlation	0.17		
Standard error	0.02		
Confidence interval, lower limit	0.13		
Confidence interval, upper limit	0.21		
Prediction interval, lower limit	0.13		
Prediction interval, upper limit	0.21		
Number of included observations	51,642		
Number of included studies	11		
Number of subgroups	2		
Analysis of variance	Sum of squares (Q*)	df	p-value
Between/Model	0.23	1	0.634
Within/Residual	9.00	9	0.438
Total	9.22	10	0.511
Pseudo R^2^	2.46%		

Discussion

This meta-analysis and systematic review demonstrates a positive, albeit modest, correlation between the risk of SSI and intraoperative bile spillage during LC. The observed r of 0.16 (95% CI = 0.07-0.25) indicates that bile spillage is a relevant, though not determinative, risk factor for postoperative infection [12]. This finding aligns with the pathophysiological rationale that bile, particularly when infected, acts as a contaminant that can trigger local inflammation and impair immune defense mechanisms [4]. These results are consistent with several large-scale observational studies included in this review, which identified bile spillage as an independent predictor of infectious complications [12,14,15].

Nevertheless, the substantial heterogeneity observed (I² = 65.01%) and the inconsistent findings across individual studies suggest that this association is not univariate. Indeed, as demonstrated in the descriptive synthesis, not all included studies reported a statistically significant increase in SSI following bile spillage, highlighting variability in reported outcomes across different populations and settings [13,18,20,22]. The variability is likely attributable to several critical moderating factors. First, the bacteriological profile of spilled bile appears to be a key determinant, as Jain et al. demonstrated that SSI rates were significantly increased only in the presence of bacteriobilia [13]. Second, the nature of the spillage itself is influential; studies by van Dijk et al. and Al-Hayali indicate that concurrent gallstone spillage may confer a higher risk of severe complications, including intra-abdominal abscess, than bile spillage alone [19,21]. These nuances help explain why other studies, particularly those with a high prevalence of sterile bile or adequate intraoperative irrigation, did not find a significant association between bile spillage and SSI [18,20].

To further explore sources of heterogeneity, performing meta-regression using surgical indication (elective vs. emergency/acute cholecystitis) was considered a potential moderator reflecting inflammatory status and bacterial load. However, this was precluded by inconsistent or absent reporting of this variable across the included studies, with several studies failing to stratify outcomes by indication or providing insufficient granularity to permit such analysis. Similarly, the potential moderating effect of intraoperative irrigation volume or technique could not be quantitatively synthesized due to heterogeneous reporting.

Subgroup analysis provides important methodological insight, demonstrating that the definition of bile spillage significantly influences the consistency of results. Studies employing a clinical or operative note-based definition (Group A) yielded a homogeneous and statistically significant effect (I² = 0%). In contrast, studies using alternative definitions (Group B) exhibited substantial heterogeneity and a non-significant pooled estimate [13,20,22]. This finding suggests that inconsistencies in exposure definition are a key contributor to variability in the literature, a phenomenon previously noted in surgical meta-analyses involving subjective intraoperative events [23].

In addition to infectious outcomes, several studies reported that bile spillage was associated with prolonged operative duration, higher conversion rates to open surgery, and extended hospital stay, suggesting that it frequently occurs in technically challenging cases characterized by severe inflammation or adhesions [12,15-17]. Notably, this synthesis challenges a common clinical practice: none of the included studies provided evidence that postoperative antibiotic prophylaxis reduces the risk of infection after bile spillage [20-22]. Specifically, studies that directly evaluated postoperative antibiotic administration demonstrated no statistically significant reduction in SSI, readmission rates, or hospital stay following bile spillage [20-22], reinforcing the lack of evidence supporting routine antibiotic escalation in this context. This observation implies that intraoperative irrigation alone may be sufficient to manage contamination and that routine postoperative antibiotic use across heterogeneous patient populations and antibiotic regimens represents an unnecessary escalation of care without demonstrated benefit in the available literature, which carries risks of adverse effects and antimicrobial resistance.

Finally, bile spillage appears to serve as a broader marker of procedural complexity. Its consistent association with longer operative times, higher conversion rates, and prolonged hospital stay across multiple studies [12,16,17] suggests that it frequently occurs in technically challenging cases characterized by dense adhesions or fragile tissues. Accordingly, bile spillage may function both as a direct contributor to adverse outcomes and as a proxy indicator of patients at higher overall surgical risk.

Limitations

First, the body of evidence is predominantly derived from observational studies, which are inherently susceptible to confounding bias. Although a random-effects model was employed, the observed associations may have been influenced by unmeasured or inadequately adjusted confounders. These include, most importantly, surgical indication (acute vs. elective cholecystitis), which directly impacts the inflammatory milieu and bacterial load; surgeon experience and laparoscopic skill level, which influence both the likelihood of spillage and the adequacy of intraoperative management; intraoperative irrigation volume and technique, which may mitigate contamination but were rarely reported in sufficient detail; and preoperative antibiotic prophylaxis status, which varied across studies and may have modified infection risk independent of spillage. Second, substantial statistical heterogeneity, despite subgroup analyses, limits the precision and generalizability of the pooled estimates. Third, evidence of publication bias, as indicated by Egger’s test, suggests that smaller studies with null findings may be underrepresented, potentially leading to an overestimation of the true effect size. Finally, variability in the definitions of both the exposure (bile spillage) and the outcome (SSI) across studies introduced clinical heterogeneity that could not be fully addressed analytically. An important limitation that prevents more precise risk stratification is the inability to perform meta-regression or subgroup analysis based on surgical indication (elective vs. emergency) or confirmed bacteriobilia status. While these factors are likely critical determinants of infection risk following bile spillage, inconsistent reporting across primary studies precluded quantitative synthesis. This limits the ability to define which specific patient subgroups carry a truly critical risk of infectious complications and represents an important gap in the literature that should be addressed in future prospective studies.

Future directions

Future research should prioritize large, prospective, multicenter cohorts employing standardized, clinically based definitions of biliary spillage, with real-time documentation of spillage content (bile vs. stones) and bile culture results. While randomizing the occurrence of spillage is not feasible, pragmatic randomized controlled trials are needed to evaluate management strategies, such as the efficacy of selective versus systematic peritoneal irrigation, the use of antibiotic-augmented irrigation solutions, and the role of targeted postoperative antibiotics only in cases of confirmed bacteriobilia.

## Conclusions

This meta-analysis demonstrates that intraoperative bile spillage during LC is associated with a modest but statistically significant increase in the risk of SSI and may also be linked to other markers of postoperative morbidity. The relationship appears to be influenced by the presence of bacteriobilia and concurrent gallstone spillage, with the latter potentially posing a higher risk for severe complications such as intra-abdominal abscess. Furthermore, methodological consistency in defining spillage substantially influences study outcomes, underscoring the need for standardized clinical criteria in future research. However, the interpretation of these findings should consider the observed heterogeneity, variability in exposure definitions, and the potential for publication bias across included studies. Importantly, current evidence provides limited support for the routine use of postoperative antibiotic prophylaxis following bile spillage, suggesting that emphasis should remain on meticulous surgical technique and thorough intraoperative irrigation rather than routine antibiotic administration.

## Appendices

**Table 6:** Comprehensive search strategy for identifying relevant studies.

All databases	Search query components	Applied filters	Syntax/Modifiers
PubMed, Embase (via Ovid), Scopus, Cochrane Central	#1 “Cholecystectomy, Laparoscopic”[Mesh] OR “laparoscopic cholecystectomy”[tiab] OR “lap cholecystectomy”[tiab]	PubMed: None for initial search	PubMed: MeSH terms, [tiab] for title/abstract
	#2 “Bile”[Mesh] OR “bile spill”[tiab] OR “gallbladder perforat”[tiab] OR “bile leak”[tiab] OR “intraoperative spill”[tiab]	Embase: ‘exp’ for explosion	Embase: ‘exp’ to include subheadings, .ti, ab,kw
	#3 “Surgical Wound Infection”[Mesh] OR “Surgical Site Infection”[tiab] OR “wound infection”[tiab] OR “port site infection”[tiab]	Scopus: TITLE-ABS-KEY() function	Scopus: TITLE-ABS-KEY(), asterisk (*) for truncation
	#4 “Postoperative Complications”[Mesh] OR “long term outcome*”[tiab] OR “survival”[tiab] OR “recurrence”[tiab] OR “adhesions”[tiab] OR “incisional hernia”[tiab]	Cochrane: No filters in Central	Cochrane: Text word search in title, abstract, keywords
	Final Query (in all databases): #1 AND #2 AND (#3 OR #4)	All: No date or language restrictions were applied initially to ensure comprehensiveness	All: Boolean operators (AND, OR), asterisk for wildcard truncation
